# Mapping and modelling the impact of mass drug adminstration on filariasis prevalence in Myanmar

**DOI:** 10.1186/s40249-018-0420-9

**Published:** 2018-05-31

**Authors:** Ni Ni Aye, Zaw Lin, Khin Nan Lon, Nay Yi Yi Linn, Thet Wai Nwe, Khin Mon Mon, Kapa Ramaiah, Hannah Betts, Louise A. Kelly-Hope

**Affiliations:** 1 Ministry of Health and Sports, Department of Public Health, Nay Pyi Taw, Myanmar; 2 Tagore Nagar, Pondicherry, India; 3 Department of Parasitology, Centre for Neglected Tropical Diseases, Liverpool School of Tropical Medicine, Liverpool, UK

**Keywords:** Lymphatic filariasis, Elephantiasis, *Wuchereria bancrofti*, Neglected tropical diseases, Mass drug administration, Transmission assessment surveys, Surveillance, Myanmar

## Abstract

**Background:**

Lymphatic filariasis (LF) is endemic in Myanmar and targeted for elimination. To highlight the National Programme to Eliminate Lymphatic Filariasis (NPELF) progress between 2000 and 2014, this paper describes the geographical distribution of LF, the scale-up and impact of mass drug administration (MDA) implementation, and the first evidence of the decline in transmission in five districts.

**Methods:**

The LF distribution was determined by mapping historical and baseline prevalence data collected by NPELF. Data on the MDA implementation, reported coverage rates and sentinel site surveillance were summarized. A statistical model was developed from the available prevalence data to predict prevalence at township level by year of measurement. Transmission assessment survey (TAS) methods, measuring antigenemia (Ag) prevalence in children, were used to determine whether prevalence was below a level where recrudescence is unlikely to occur.

**Results:**

The highest baseline LF prevalence was found in the Central Valley region. The MDA implementation activities scaled up to cover 45 districts, representing the majority of the endemic population, with drug coverage rates ranging from 60.0% to 98.5%. Challenges related to drug supply and local conflict were reported, and interrupted MDA in some districts. Overall, significant reductions in LF prevalence were found, especially after the first 2 to 3 rounds of MDA, which was supported by the corresponding model. The TAS activities in five districts found only two Ag positive children, resulting in all districts passing the critical threshold.

**Conclusion:**

Overall, the Myanmar NPELF has made positive steps forward in the elimination of LF despite several challenges, however, it needs to maintain momentum, drawing on international stakeholder support, to aim towards the national and global goals of elimination.

**Electronic supplementary material:**

The online version of this article (10.1186/s40249-018-0420-9) contains supplementary material, which is available to authorized users.

## Multilingual abstracts

Please see Additional file 1 for translations of the abstract into the five official working languages of the United Nations.

## Background

Lymphatic filariasis (LF) is a major public health problem in tropical and sub-tropical countries due to the painful, disabling and disfiguring clinical conditions associated with chronic infection [1, 2]. The disease is caused by infection with filarial worms and transmitted by a range of mosquito species. In humans, the infective filarial larvae target the lymphatic system, grow to become adult worms and reproduce causing conditions such as lymphoedema (swelling of arms, legs or breasts), and hydrocoele (scrotal swelling) in men [3, 4]. The South-East Asia region accounts for the highest burden of disease in the world [5–7], and many countries adopted the strategy of the Global Program to Eliminate Lymphatic Filariasis (GPELF) which comprises the main goals of i) interrupting transmission through at least five annual rounds of mass drug administration (MDA) with 65% coverage of total population, and ii) alleviating suffering through morbidity management and disability prevention (MMDP) through the provision of a package of care to manage lymphedema and hydrocoele within primary health care systems [8].

Myanmar, formerly known as Burma, is one of the most endemic countries in South-East Asia, with a high LF prevalence, where the disease is caused by the parasite *Wuchereria bancrofti* and transmitted by the mosquito *Culex quinquefasciatus* [9, 10]*.* Like many other countries in the region, Myanmar has a long history of filarial endemicity, with high infection prevalence levels in several foci [7, 9, 10]. The Myanmar government therefore responded to the new GPELF programme, and developed the National Programme to Eliminate Lymphatic Filariasis (NPELF) in 2000. The Myanmar NPELF drew on the historical evidence, national data and mapping studies conducted in the late 1990s to demarcate the endemic districts of the country [11]. The peninsular and central inland areas were found to be most endemic with an estimated 41 million people (~ 80% of total population) to be at risk of infection in 45 districts.

The initial primary focus of Myanmar NPELF was to interrupt transmission by reducing prevalence rates through MDA using two anti-filarial drugs; diethylcarbamazine (DEC) and albendazole. Over the past 15 year, the NPELF has been up-scaling and down-scaling programmatic activities, including developing a National LF elimination plan for the WHO in 2000, starting MDA implementation in 2001 and reaching 43 districts in 2013, conducting ongoing sentinel site surveillance since 2000, and implementing the first surveys to show evidence of impact and reductions in prevalence in 2008 and 2014 using standard World Health Organization (WHO) guidelines [12–14]. Overall, key steps forward have been made, despite several challenges related to the availability of funding, and ready access to the drug DEC. To highlight the programmatic activities in Myanmar, this paper describes and maps the geographical distribution of LF, outlines the progress and impact of programmatic activities, models the decline in prevalence, and highlights the first evidence that prevalence has been lowered to an extent where transmission is likely to be no longer sustainable in five districts.

## Methods

### Study area

**Fig. 1:**
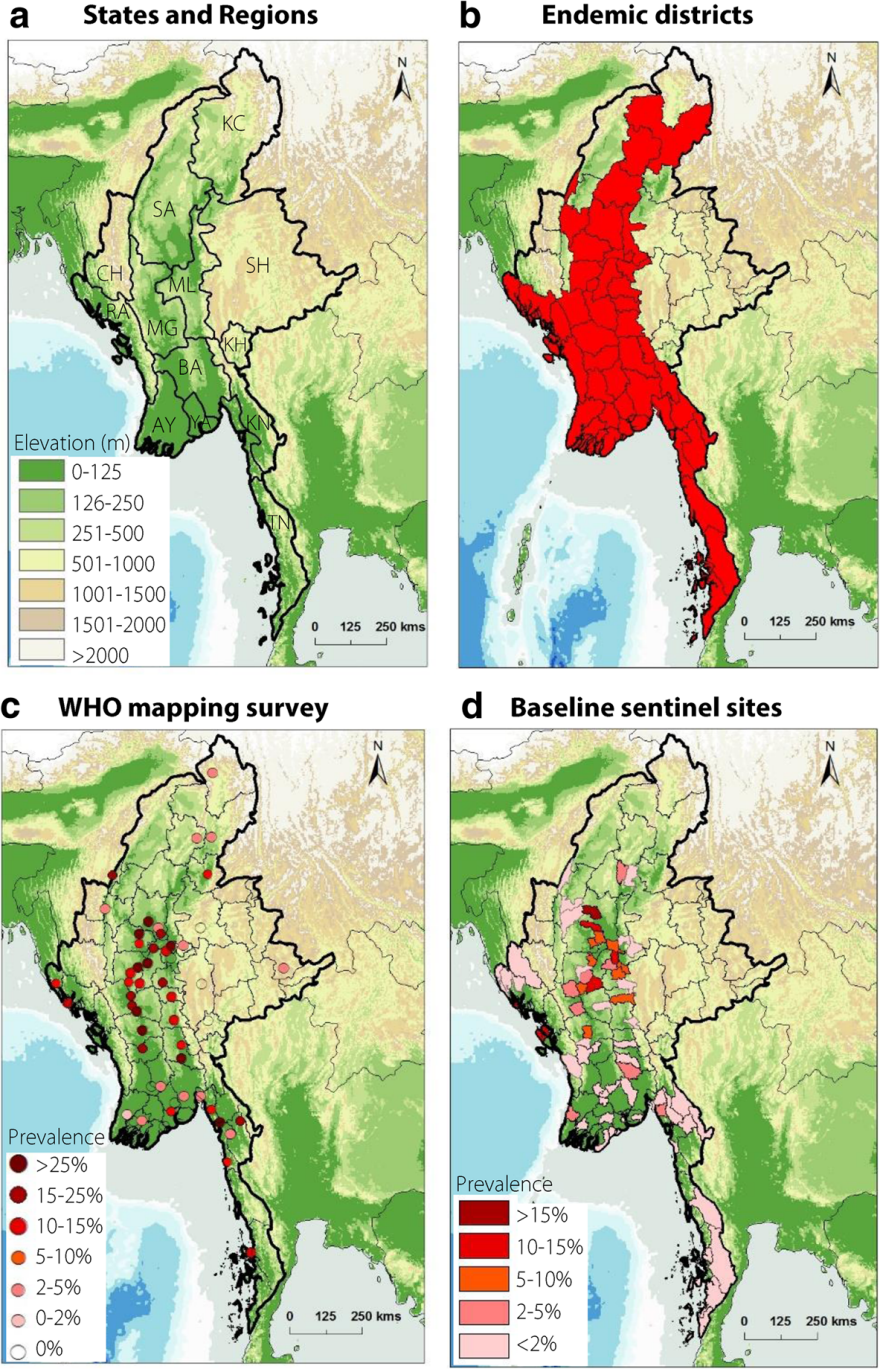
Map of administrative units and distribution of LF endemicity before intervention. Note: (**a**). State and Region abbreviations: Kachin (KC); Sagaing (SA), Chin (CH), Shan (SH), Mandalay (ML), Magway (MG), Rakhine (RA), Ayeyarwady (AY), Yangon (YA), Bago (BA), Kayah (KH), Kayin (KN), Mon (MO), Tanintharyi (TN), (**b**) Endemic districts, (**c**) Prevalence based on antigenaemia (Ag) determined using the immuno-chromatographic test (ICT) card (BinaxNOW Filariasis, Alere Inc.,) kits. **d** Prevalence based on microfilaria (Mf) survey data

Myanmar is a lower middle income tropical country, and divided into seven states, and seven regions with a capital NayPyiTaw Union Territory established in 2005 (Fig. 1a). These 15 administrative areas are further organized into districts, townships, towns, cities, wards, village-tracts (groups of adjacent villages) and villages. The latest census data in 2014 indicated that Myanmar has a population of 51.5 million, with a population density of 75 per square kilometre, and more than one third of the population living in urban areas [15]. Topographically, there are three distinct regions, which include the Western Hill Region, Eastern Hill Region and the Central Valley Region dominated by the Ayeyarwady basin with low elevation levels.

The NPELF is part of the Ministry of Health and Sports (MoHS) and responsible for the MDA implementation and MMDP activities across the country [16]. In 2000, Myanmar had a total of 65 districts, which the NPELF continues to use for programmatic purposes, despite the recent changes in administrative boundaries to form 74 districts in 2014. The MoHS delivers the preventive and curative health services at all levels, including the LF Programme. Each sub-rural health centre provides health-care services to a cluster of five to ten villages, which have health volunteers and who also assist with the LF MDA activities as community drug distributors.

### Geographical distribution of LF

The endemicity status of each of the 65 districts in Myanmar in 2000, was based on collated historical data, national reports and rapid prevalence mapping surveys conducted in 19 districts as part of a WHO multi-country study [11]. Based on this, 45 districts out of the 65 districts were defined as endemic. The most extensive mapping survey conducted by the WHO included a total of 70 randomly selected townships in the 19 districts. The prevalence of antigenaemia (Ag) was determined using the immuno-chromatographic test (ICT) card (BinaxNOW Filariasis, Alere Inc., Scarborough, ME) antigen detection kits from 100 voluntary participants from randomly selected wards and households, which included everyone in each household except the very ill and those people who were not present at the time of survey.

To highlight the endemic distribution across the country before the scale up of MDA related activities, the WHO prevalence point data were re-mapped across the 45 endemic districts by importing the original map and digitizing the points using a standard point feature tool in the geographical information system software ArcGIS 10 (ESRI, Redlands, CA). The Global Digital Elevation Model (ETOPO2) was used as a base map, which was available from ESRI, Redlands, CA.

### MDA implementation and sentinel sites

To describe the progress and impact of activities related to the decline in transmission, data on the MDA implementation, reported coverage and sentinel site surveillance (including randomly selected spot check sites) were summarised. The MDA implementation activities were conducted in accordance with the GPELF strategy where each district, also known as the implementation unit (IU), is required to conduct at least five rounds of MDA, with > 65% coverage rate of the entire population [13]. MDA is community-based and implemented using the directly observed treatment practice via a door-to-door or booth distribution by community volunteers, once a year over the period of a week. The main social mobilization activities implemented in communities before MDA included televised media, radio broadcasting, health talks in the community by programme staff and basic health staff (i.e. health assistants, midwives, trained nurses), and the distribution of pamphlets with the assistance from civil society organizations and local authorities. A protocol was developed on how to report and respond to a range of adverse reactions before the start of the MDA advocacy, with the basic health staff trained on the procedures.

The monitoring and evaluation of the programme was conducted through regular sentinel and spot check site surveillance at township level. These field data were analysed to help assess the impact before- during- after MDA implementation activities. Standard surveillance involved selecting two sites (villages) per IU, with between 300 to 500 people (including all ages ≥2 years of age), selected for assessment of infection by examining night bloods slides for microfilaria (Mf) as per standard guidelines [13]. The blood smears were processed and examined in each IU (district) headquarters. The data were then sent to the central level, where all the data were maintained in registers and examined at township level. All baseline sentinel site prevalence data were mapped by Township geographical boundary using the software ArcGIS 10 (ESRI, Redland, CA).

### Modelling the impact of MDA on prevalence

To better understand the reductions in prevalence associated with MDA, a statistical model was developed to predict the change in Mf prevalence in a township since the most recent assessment of prevalence, which varied by site. This most recent Mf value was therefore referred to below as the “most recent Mf value”. Data were filtered to include only townships for which both a baseline Mf value (i.e. measurement taken prior to any MDA), and at least one later sentinel and/or spot check Mf value were available. Where there were multiple Mf values recorded at the same township in the same a year, a mean value was taken and used. Variables considered for the model included the baseline Mf prevalence value, the most recent Mf value (this may have been either a baseline Mf prevalence value or the results of a post-baseline programmatic survey), the number of MDA rounds undertaken since the beginning of the MDA programme, and the number of MDA rounds and years since the most recent Mf value was collected. Also considered were calculated variables that attempted to quantify the fragmented nature of the MDA programme; for example, the number of MDAs since the most recent Mf value divided by the number of years over which these MDAs has been administered. Also, the total number of MDAs since the beginning of the MDA programme divided by the number of years over, which the programme was administered. Finally, the maximum and mean number of years between MDAs, both since the start of the programme and since the most recent Mf value.

First, all Mf values were transformed using log10(x + 1) due to asymmetry observed in qq plot when untransformed data was modelled. A number of other transformations were also considered. A Generalized Linear Model (GLM, function ‘glm’) within the R statistical environment (R Development Core Team, 2012) was then used to predict the average Mf values for each township in a year.

The parsimony protocol outlined by Crawley [17] was used to simplify the model by removing any redundant variables and producing the Minimum Adequate Model (MAM), i.e. non-significant values and interaction terms were removed sequentially from the highest order interactions downwards. At each step, the significance of deleted items was assessed using analysis of variance using the AIC statistic.

### Interruption of transmission

The transmission assessment survey (TAS) is a standardised decision-making tool developed and recommended by the WHO [13], and was used to determine the decline in transmission in 2014 in five districts from three regions, including the Magway Region (Minbu District), Sagaing Region (Kathur, Kalay, Tamu Districts), and Mandalay Region (Pyin Oolwin District). Prior to stopping MDA, each district had at least five effective rounds of MDA, showed evidence of > 65% coverage rates, and demonstrated significant reduction in Ag (< 2%) and Mf (< 1%) prevalence rates in all sites.

The Kathur, Kalay and Tamu Districts stopped MDA in late 2007, and were assessed for the decline in transmission using cluster surveys in 2008 according to WHO guidelines at the time. Therefore, the TAS surveys conducted in 2014 were considered to be the second TAS or “TAS 2” for these three districts. The results of the first cluster surveys or “TAS 1” from the 2008 surveys, and the results from TAS 2 in 2014 were presented for these three districts.

The TAS survey design was dependent upon factors such as the net primary school rate in each evaluation unit (EU), the target population size, school enrolment, number of schools, mosquito vector type and parasite species. The TAS Survey Sample Builder was used to automate the calculations of the sample design, size, intervals and critical cut-off values. The rapid ICT Binax NOW Filariasis (Alere Inc., Scarborough, ME) was used to detect circulating filarial antigen (CFA) in the children, and validated with a positive control prior to the commencement of the survey.

## Results

### Distribution of LF

The WHO LF prevalence survey found filarial antigen ranged from 0% to > 25%. These data were analysed spatially to provide an estimated prevalence of filarial antigen for each district, which highlighted the major filarial focus in the central region of the country. Based on this national reports and historical data, the NPELF defined the 45 endemic IUs (districts) that required MDA, which were predominately in the lowland areas in the Central Valley Region (Fig. 1b and c).

The baseline Mf sentinel site prevalence for each IU was conducted prior to MDA implementation. Sentinel sites were conducted in a step-wise manner over a 13 year period between 2001 and 2013. Table 1 summarises the MDA and Mf sentinel site information for endemic IUs in each region. All baseline sentinel site prevalence data were mapped by township geographical boundary, which highlighted similar endemicity patterns in the Central Valley Region (Fig. 1d).

**Table 1:** Summary of regional/provincial endemic districts, MDA start dates and sentinel site prevalence

Region/ Province	Endemic Districts (Implementation Unit – IUs)	Year of MDA Start	Year of Baseline Sentinel Site	Mf^a^ Baseline Average (no. sites)	Mf Baseline Range	Mf ^b^3–5 MDA Round Average (no. sites)	Mf ^b^ > 5 MDA Round Average (no. sites)
Magway	Magway, Thayet, Minbu, Pakoku,	2001–2002	2001–2002	3.2 (*n* = 9)	0.2–9.1	2.3 (*n* = 21)	1.2 (*n* = 29)
Sagaing	Sagaing, Shwe Bo, Monywa, Kathur, Kalay, Tamu,	2002	2002	4.7 (*n* = 11)	0–15.1	2.8 (*n* = 12)	1.2 (*n* = 43)
Chin	Paletwa	2004	2003	1.5 (*n* = 2)	1.4–1.6	0.2 (*n* = 2)	-
Mandalay	Mandalay, Pyin Oo Lwin, Kyauk Se, Yamethin, Myin Gyan, Meikhtilar, Nyaung Oo	2004	2003	5.2 (*n* = 14)	0.2–14.7	-	2.3 (*n* = 20)
Nay pyi taw	Nay pyi taw	2013	2011	1.9 (*n* = 1)	1.9	-	-
Rakhine	Sittwe, Maungdaw, Kyauk Phyu, Thandwe	2004	2003–2004	3.0 (*n* = 6)	0–12.6	0.6 (*n* = 13)	
Ayeyarwaddy	Pathein, Hantada, Myaungmya, Phyarpone. Maubin	2013	2002–2013	0.5 (*n* = 12)	0–2.4	-	-
Bago	Bago, Thanung Ngu, Tharyawaddy, Pyay	2013	2005–2013	0.7 (*n* = 10)	0–2.8	-	-
Kayin	Hpaan, Kawkareik, Myawaddy	2013	2013	0.002 (*n* = 5)	0–0.1	-	-
Mon	Mawlamying, Thaton	2013	2004–2013	1.8 (*n* = 4)	0.8–3.2	-	-
Tanintharyi	Dawei, Myeik, Kawthaung	2013	2004–2013	0.5 (*n* = 8)	0–1.8	-	-
Yangon	Yangon North, Yangon South, Yangon East, Yangon West	2013	2004–2013	0.1 (*n* = 8)	0–0.4	-	-

^a^Mf average based on the total number of sites (in brackets) across the endemic districts. At each site between 300 and 500 people tested. Some sites were tested multiple years before MDA started

^b^Mf based on both sentinel sites and spot check sites based on number of sites (in brackets). At each site between 300 and 500 people tested

Mf: microfilaria

The Mf baseline average rates were highest in Sagaing (7.9%), Mandalay (5.2%) and Magway (3.6%) Regions with the highest rates recorded in the districts of Shwe Bo (15.1%), Kyauk Se (14.7%) and Pakokku (9.1%) in 2002, 2003 and 2002, respectively. Overall the Mf baseline average rates were lowest in the districts of Ayeyawaddy (0.5%), Kayin (0.002%) and Tanintharyi (0.5%) Regions with the highest Mf rates recorded in Pathein (2.4%), Myawaddy (0.1%) and Dawei (1.8%) in 2004, 2012 and 2008, respectively. See Additional file 2 for baseline sentinel site prevalence data.

### MDA implementation

**Fig. 2:**
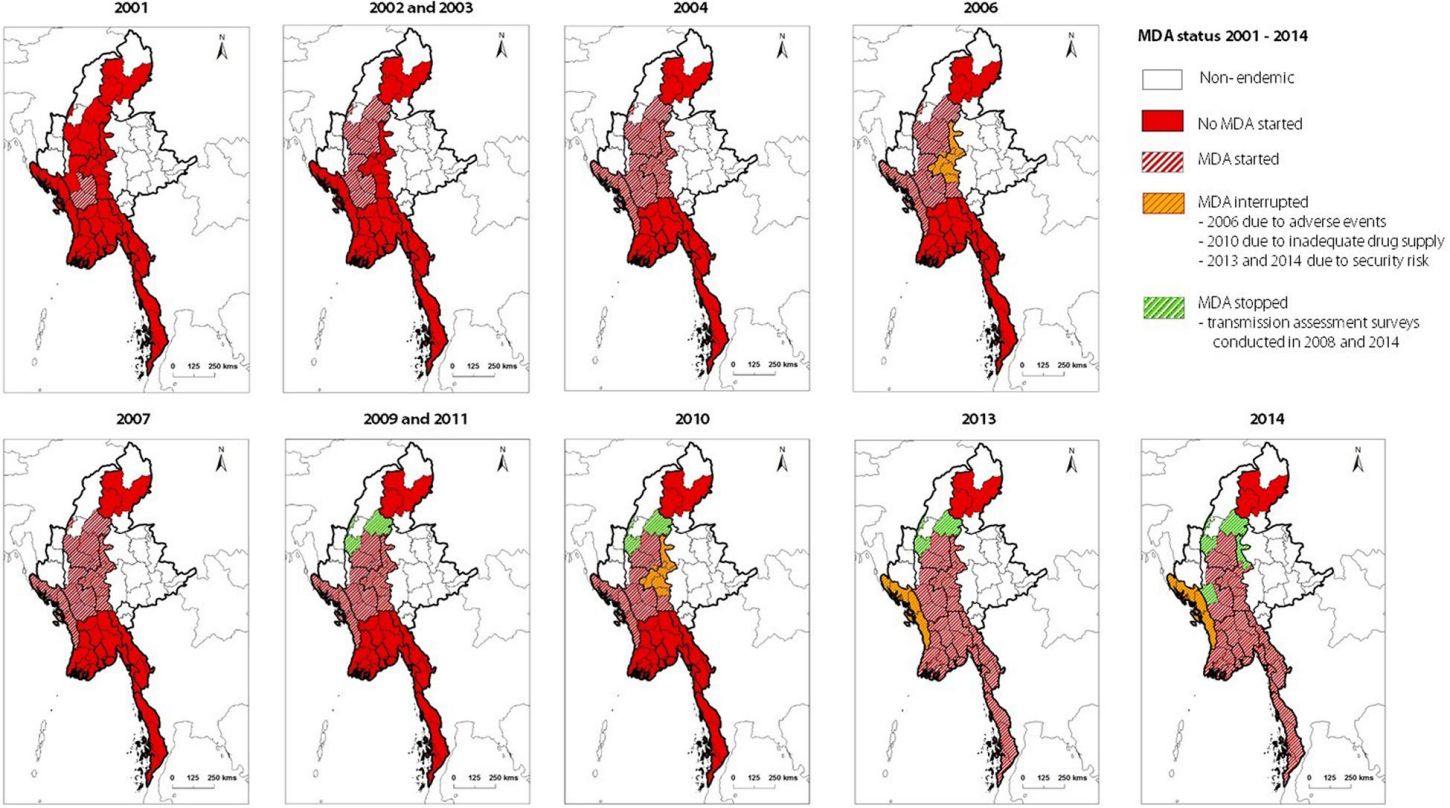
Programme MDA up scale and down scale between 2001 and 2014

The first MDA was implemented in two IUs viz., Magway and Theyet Districts in the Magway Region in 2001 (Fig. 2). MDA was extended to 10 IUs across the Magway and Sagaing Regions in 2002, and then to 22 IUs in 2004, representing approximately 48% geographical coverage. The majority of these MDA IUs had a relatively higher burden of LF. The NPELF aimed to implement MDA uninterruptedly, however, no MDA took place in the years 2005 and 2008 due to delays in DEC supplies from the donors. Further, in the Mandalay Region, seven IUs had MDA interrupted twice over the study period; in 2006 due to incidence of serious adverse reactions during the preceding MDAs, and in 2010 due to DEC supply constraints. The adverse reactions observed during 2001–2004 after the initial MDAs were conducted in Magway Region included giddiness, headaches, nausea, rashes, fever, urticaria and vomiting. This is the only data on adverse reactions available to present, and it is recognized the reporting system needs to be strengthened.

The expansion of MDA activities did not take place until 2013. Considerable efforts were made to extend the MDA programme to cover all endemic IUs in 2011 and 2012. However, the NPELF could not scale up due to the lack of funding, need for many resources (e.g. training of basic health staff, advocacy materials), continued problems in procuring adequate quantities of DEC, as well as security related issues in the two endemic districts of Kachin State. No MDA was conducted in 2012. However, in 2013, a major increase in coverage was finally reached with support from Sanofi Pasteur, WHO and the Global Network for NTDs for supply of DEC and operational costs. This up scaling of MDA resulted in a further 21 IUs receiving treatment in 2013, which coincided with the down scaling of MDA in 2 IUs (Minbu and Pyin Oo Lwin Districts) as they reached the requirements for TAS, and a further 4 IUs from Rakhine State temporarily stopping MDA due to security related issues. Additional file 3 summarises the scale up and scale down of MDA related activities between 2001 and 2014.

Overall, the reported treatment coverage i.e. the coverage calculated for each IU on the basis of reports sent from lower level units (townships, villages) was high, ranging from 68.7% to 98.5% of the IU’s entire population (Table 2). A cross-sectional evaluation of the treatment coverage conducted by a team within the Department of Health in randomly selected townships in 2013, found that coverage rates ranged from 60% to 97.4% across 19 IUs, and reflected reported coverage rates in each corresponding IU. Of those people who were eligible, but did not take the drug, the main reasons were that they were absent at the time of MDA, or had refused to take the drugs related to negative rumours regarding MDA.

**Table 2:** Summary of population and reported treatment coverage rates 2001–2014

Year	No. of regions	No. of districts	Total population covered	Population which ingested drugs	Reported Treatment coverage	
					Overall	Range among districts
2001	1	2	1 939 964	1 803 306	93	-
2002	2	10	8 634 179	7 474 094	95.7	93.2–98.5
2004	5	22	17 929 178	15 838 896	82.6	80.1–94.3
2005	-	-	No MDA	No MDA	-	-
2006	4	15	11 868 901	10 761 777	90.7	79.5–94.1
2007	5	22	20 000 250	18 397 240	91.9	83.4–93.9
2008	-	-	No MDA	No MDA	-	-
2009	5	19	17 702 845	15 790 286	89.2	85.5–96.4
2010	4	12	10 035 458	9 002 092	89.7	82.7–95.4
2011	5	19	1 7031 636	15 429 100	90.5	85.8–93.4
2012	-	-	No MDA	No MDA	-	-
2013	7	36	35 488 298	30 313 249	85.4	68.7–93.8
2014	7	37	36 407 716	31 121 035	85.5	74.8–93.7

### Impact of MDA on field collected prevalence data (baseline and sentinel/spot check sites)

**Fig. 3:**
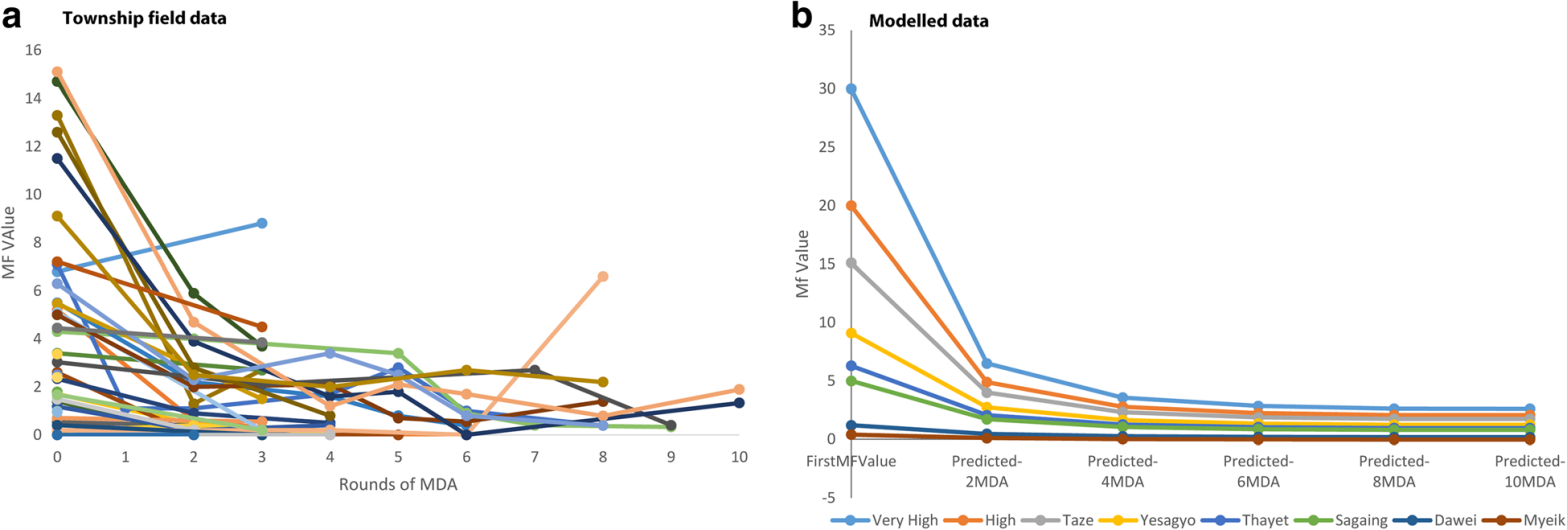
Sentinel and spot check data and modelled distributions by number of MDA rounds. (**a**) Township data (**b**) Modelled data. Note. Township data includes all points from all sentinel sites and modelled data include selected sites

The field collected baseline data varied significantly between 15.1% to zero in 46 townships across the country. Figure 3a highlights the prevalence trends by the number of MDA rounds. All but two townships displayed a significant reduction in prevalence over the 13 years for which data was available, especially after two to three MDA rounds where the prevalence ranged from 0 to 8.8%. The two townships, Amapura and Pakokku, which reported a rise in prevalence at spot check sites (outliers) were removed so that general trends could better be examined. This resulted in 138 individual Mf site values included in further analysis, and showed that the prevalence after two to three MDA rounds was significantly reduced, and ranged from 0 to 5.9%.

### Modelling the impact of MDA on prevalence

The townships for which sentinel site and spot check prevalence data were available received between 0 and 12 rounds of MDA. A model predicting the Mf prevalence in a township in a year from the most recent assessment of Mf prevalence at the same township was produced. After the removal of redundant terms, the model consisted of two coefficients (Fig. 3b; Table 3). The most significant coefficient, was an interaction term consisting of two predictor variables (the most recently collected Mf prevalence (log + 1) and the number of MDAs administered since that Mf prevalence data were collected (log + 1). The second most significant coefficient was the baseline prevalence (log + 1). Together, this demonstrates that whilst the number of rounds of MDA is the most important predictor, the underlying conditions reflected by the baseline figure also affects the results, i.e. two rounds of MDA in a high baseline area will not reduce prevalence as much as two rounds of MDA in a low baselines area, even if the most recent Mf value in the two areas is the same. Surprisingly, the covariates that described the fragmented nature of the MDA (i.e. the number of missed MDA years) did not feature in the MAM. That is, they did not significantly improve the fit of the model. This may be due to the small number of data points available.

**Table 3:** Minimal Adequate Model (MAM) predicting prevalence at Township level from prevalence previously measured and number rounds MDAs

Coefficients:	Estimate	Std. Error	*t* value	Pr(>|t|)
(Intercept)	- 0.05012	0.03641	- 1.377	0.172
logBaselineMf	0.27098	0.05263	5.149	1.77e - 06
logPreviousMf: logMDAsSincePreviousMf	0.73239	0.11677	6.272	1.59e - 08

Residual standard error: 0.1507 on 82 degrees of freedom

Multiple R-squared: 0.6162, Adjusted R-squared: 0.6069

F-statistic: 65.84 on 2 and 82 DF, *P*-value < 2.2e - 16

The model unexpectedly predicted an increase in prevalence if there were more rounds of MDA between measurements of prevalence. However, this appears to be an artifact of the MDA administration; prevalence data were usually collected after every two MDA rounds but were collected after 3 rounds of MDA in Mandalay. The model shows that three rounds of MDA in Mandalay had less effect on prevalence than two rounds of MDA elsewhere; therefore suggesting that in Mandalay specifically, the MDAs were less effective.

The modelled data shown in Fig. 3b clearly reflects the same prevalence trend as the field collected data. The initial two MDA rounds produced a significant reduction in prevalence which was followed by a much smaller reduction in subsequent MDA rounds, with prevalence levelling out. The models suggest a similar trend, for example an area with 30% baseline prevalence, drops to 6.5% after the first two MDA rounds indicating a reduction of 78%. The next two MDA rounds reduce further, but less dramatically, to 3.5%, and two further MDA rounds reduce prevalence to 2.8%. For sites with a baseline of 15%, the effect of the first two MDA rounds is a 73% reduction to 3.9% prevalence (next two MDA rounds reduce prevalence to 2.3%, with little further effect after four MDA rounds). For areas with a baseline of 7%, the effect of the first two rounds of MDA is a 69% reduction to a prevalence of 2.2% (next two MDA rounds reduce to 1.4% prevalence, with little effect after four MDA rounds).

### Decline in transmission

In 2008, the first cluster surveys or “TAS 1” for the Kalay, Kathar and Tamu Districts found no ICT positive children among the 2269, 3003, and 3085 tested across 16, 31, and 25 schools respectively. Further details of the schools surveyed are in Additional files 4, 5, and 6.

In 2014, the pre-TAS assessments indicated that all five IUs had sufficient number of MDA rounds, and high reported treatment coverage rates of > 85%. Based on the population size and school enrolment rates of > 90%, school-based surveys were conducted in each IUs, which were evaluated as EUs, with sample sizes of between 1556 to 1548 children across 30 to 52 schools, and critical cut-off of 18 positive children calculated for all EUs (Table 4).

**Table 4:** Summary of IU populations, MDA and TAS characteristics

	Population and MDA			TAS					
Districts	Total Pop.	MDA Rounds & Years	MDA Coverage Range	No. 6–7 aged Children	No. Schools	Total Sample Size	No. Schools Tested	Children Absent %	No. ICT Positive
**Minbu** TAS 1	689 965	10 MDAs 2002–2014	86–94%	27 252	759	1556	52	2.3%	1
**Pyin Oolwin** TAS 1	851 945	6 MDAs 2004–2014	85–99%	32 529	634	1556	36	0.2%	0
**Kalay** TAS 2	487 762	5 MDAs 2002–2007	85–98%	24 461	387	1556	30	0.7%	0
**Kathar** TAS 2	819 281	5 MDAs 2002–2007	92–100%	34 090	1077	1556	58	0.4%	1
**Tamu** TAS 2	105 100	5 MDA 2002–2007	92–100%	17 155	77	1548	30	0%	0

Note. Details of MDA years available in Additional file 3

IU: Implementation Unit

MDA: Mass drug administration

TAS: Transmission assessment survey

The field activities involved 5 to 7 teams consisting of three people each: supervisor, data collector and a technician. All team members were trained per the TAS guidelines by a Central and Regional Team leader. The Township Medical Officer was responsible for informing and coordinating activities with the Township Education Department, and Headmasters of the selected school before the survey. The Headmaster of each school provided an official class register and all eligible children were identified for selection. For each selected child, their name, sex, age, and grade was recorded and 100 μl of blood collected for ICT. No child refused to participate in the survey.

The TAS 1 results for Minbu and Pyin Oo Lwin EUs found 1 positive child, and the TAS 2 results for Kalay, Kathar and Tamu EUs found 1 positive child (Table 4). These results resulted in all EUs being under the critical cut-off and passing TAS. The two positive children were treated and the parents and relative Township Medical Officer informed for further monitoring as required.

## Discussion

Overall good programmatic progress has been made by the Myanmar NPELF with the successful scale up of MDA implementation, widespread significant reductions in prevalence, and the initiation of post-MDA surveillance activities in five districts. This national overview extends the findings from four endemic states/regions by Win et al., and highlights that progress has been achieved despite some challenges in obtaining regular funding and support from international partners and stakeholders, reports of side-effects during MDA, internal security issues, and difficulties in obtaining the drug DEC. The progress to-date may be attributed to several factors identified as determinants of LF programme success as noted in other countries [18, 19] including the i) general low transmission levels found at baseline with the majority of Mf rates < 15%; ii) MDA regime of albendazole and DEC which is considered a highly effective combination against the parasite *W. bancrofti*; and iii) good health system infrastructure, administration and training. It is also likely that this widespread MDA coverage with albendazole for LF has impacted on soil transmitted helminths (STH) infection rates, which will be beneficial to the STH MDA programme and should be taken into account [20].

Notwithstanding these positive steps forward, the NPELF will face some challenges to complete all programmatic activities by the GPELF elimination goal of 2020 [21, 22]. It will require pro-active interaction with drug donors to ensure steady DEC supply. International collaboration and support may be more achievable now with the changed political situation in country [23]. This may help to address any challenges that arise and maintain the momentum of the programme. The NPELF is at crucial stage now in terms of undertaking multiple activities in as many as 45 districts. Hence, more technical and financial support from international partners and stakeholders is key to its future success.

Importantly, MDA implementation was initially focused in high transmission areas, and the programme was able to demonstrate that the most significant reductions occurred after 2 to 3 rounds of MDA. This transmission reduction pattern is consistent with other studies in the South-East Asian Region and elsewhere [19, 24–27], and is in agreement with the predicted model presented in this paper. The model confirms that LF prevalence reduces with number of MDAs. However, the effect of each subsequent MDA is less than the one before, and also dependent on the initial prevalence rate. Notably, after a number of MDAs, the prevalence reaches an asymptote i.e. levels out to a point, that was proportional to the initial prevalence rate. This suggests that, whilst in low endemic areas MDA may be sufficient to reach elimination, in higher endemic areas, reducing the transmission levels to zero may prove difficult and the current MDA strategy would potentially benefit from supplementary interventions such as vector control, and environmental management to better control the *Culex* spp. vectors [28]. Thus, information on the vector biting rates may be key for better insights into the transmission dynamics and elimination in a geographic region, especially in the endgame phase where there is the risk of recrudescence, as highlighted in recent mathematical models [29, 30].

The NPELF may also consider the possibility and feasibility of triple drug therapy, including ivermectin, DEC and albendazole (IDA) and the protocols have recently been released for field use [31, 32]. The high effectiveness of this therapy, may shorten the duration and cost of MDAs, especially in potential ‘hotspot’ areas, or where MDA coverage has been repeatedly interrupted such as Mandalay, which had only three MDAs over a 8 year period and showed areas of persistent infection [33]. The IDA may also be useful for areas with low, or lowered prevalence where transmission appears difficult to ultimately interrupt [28]. However, measuring and modelling the impact of IDA on filariasis prevalence will be crucial. Understanding the barriers to high MDA coverage will also be important, and more in-depth studies in problem areas should be undertaken [34, 35]. Further, it will be important for the NPELF to strengthen its response to and recording of adverse reactions as this has been a programme weakness to date.

The TAS in five districts confirms that transmission has declined significantly in some areas of the country. However, as standard post-MDA surveillance activities scale up over the next 5 years, the NPELF could be further strengthened by training more personnel and increasing the number of teams to support the activities on a more full-time basis as other countries have done [7, 19]. Integrating alternative methods of monitoring and evaluation into the existing health system structure will also be important to ensure that surveillance is sustainable long-term, and sufficiently sensitive and targeted to find potential problem areas or hotspots of transmission [36, 37]. In parallel, MMDP mapping and related activities, and additional integrated post-TAS activities need be initiated and could be conducted using new tools and field scenarios [7, 38]. The districts with high burden of chronic disease and high baseline infection should be prioritized, and the key activities integrated with health system.

## Conclusions

This study highlights that the Myanmar NPELF has made positive steps forward in the elimination of LF with significant reductions in prevalence and the first evidence of interrupting transmission. It will be important for the NPELF to maintain this momentum, aim to maximize its capacity and draw on international stakeholder support to help meet the national and global goals of elimination.

## Additional files

Additional file 1: Multilingual abstracts in the five official working languages of the United Nations. (PDF 250 kb)

Additional file 2: Baseline Mf prevalence in district sentinel sites. (DOCX 35 kb)

Additional file 3: Matrix of MDA in endemic districts from 2001 to 2014. (DOCX 37 kb)

Additional file 4: ICT Survey in school children in Tamu Township District in 2008. (DOCX 23 kb)

Additional file 5: ICT Survey in school children in Katha District in 2008. (DOCX 25 kb)

Additional file 6: ICT Survey in infant and school children from Kalay District in 2008. (DOCX 22 kb)

## Abbreviations

Ag: Antigenaemia
DEC: Diethylcarbamazine
EU: Evaluation unit
ICT: Immuno-chromatographic test
IU: Implementation unit
GIS: Geographical information system
GPELF: Global program to eliminate lymphatic filariasis
LF: Lymphatic filariasis
MDA: Mass drug administration
Mf: Microfilaria
MAM: Minimum adequate model
MoHS: Ministry of Health and Sports
MMDP: Morbidity management and disability prevention
NPELF: National Programme to Eliminate Lymphatic Filariasis
TAS: Transmission assessment survey
WHO: World Health Organization
