# Supplementary material for: Mapping and modelling the impact of mass drug adminstration on filariasis prevalence in Myanmar
Source: Infect Dis Poverty. 2018 May 31;7:56. doi: 10.1186/s40249-018-0420-9 (PMC5984392; doi:10.1186/s40249-018-0420-9)
Supplement: Supplementary file 1 — Multilingual abstracts in the five official working languages of the United Nations. (PDF 250 kb) [file 40249_2018_420_MOESM1_ESM.pdf]

Translation of the abstract into the five official working languages of the United Nations

داء الفيلاريات اللمفي والتقدّم المبرمج للقضاء عليه في ميانمار بين عامي 2000 – 2014

ني ني أي، زاو لين، خين نان لون، ناي يي يي لين، ثيت واي نوي، خين مون مون، كابا راميا، هانا بيتس، لويز إي كيلي-هوب

الملخص

خلفية: داء الفيلاريّات اللمفي مرض متوطن في ميانمار والقضاء عليه امر هدفي لتسليط الضوء على تقدّم البرنامج الوطني للقضاء على داء الفيلاريات اللمفاوي (NPELF) بين عامي 2000 - 2014، تسلط هذه الورقة الضوء على التوزيع الجغرافي لداء الفيلاريات اللمفي، وتوسيع نطاق الإعطاء الجموعي للدواء (MDA) وتأثيره، وأول دليل على الانخفاض في انتقال المرض في خمس مناطق.

الطرق: حُدّد توزع داء الفيلاريات اللمفي عن طريق وضع خرائط بيانات الانتشار الأساسية والتاريخية التي جُمعت من قبل البرنامج الوطني للقضاء على داء الفيلاريات اللمفاوي ولُخّصت البيانات المتعلقة بتنفيذ الإعطاء الجموعي للادوية، ومعدلات التغطية المبلغ عنها ومراقبة الترصّد المخفري. ثم طُوّر نموذج إحصائي من بيانات الانتشار المتاحة للتنبؤ بانتشاره على مستوى البلديات حسب سنة القياس. استُخدمت طرق مسح تقييم الانتقال (TAS)، وقياس انتشار وُجودُ المُسْتَضِدَّاتِ فِي الدَّم (Ag) لدى الأطفال، لتحديد ما إذا كان الانتشار أقل من المستوى الذي لا يرجح معه حدوث الأيبويّة (تكرر الإصابة).

النتائج: عُثر على أعلى معدل أساسي لانتشار داء الفيلاريات اللمفي في منطقة الوادي المركزي. وارتفع حجم أنشطة تطبيق إعطاء الأدوية الجموعي لتشمل 45 مقاطعة، تمثل غالبية المجموعات السكانية التي سيتوطن المرض بينها، وتراوحت معدلات التغطية الدوائية بين 60٪ و 98.5٪. ذكر حدوث صعوبات ذات صلة بإمدادات الأدوية والنزاعات المحلية، وتوقّف إعطاء الأدوية الجموعي في بعض المناطق. بشكل عام ، لوحظ انخفاض ملموس في انتشار داء الفيلاريات اللمفي، لا سيما بعد أول دورتين – ثلاث دورات من إعطاء الأدوية الجموعي، وهذا ما تم دعمه عن طريق النموذج المقابل. لم تعثر أنشطة مسح تقييم الانتقال إلا على طفلين إيجابيي المستضدات في الدم، وكانت النتيجة تجاوز جميع المناطق الخمس العتبة الحرجة.

الخلاصة: بشكل إجمالي، خطى البرنامج الوطني للقضاء على داء الفيلاريات اللمفاوي في ميانمار خطوات إيجابية إلى الأمام في القضاء على داء الفيلاريات اللمفي على الرغم من وجود عدد من الصعوبات، بيد أنه بحاجة للحفاظ على الزخم، بالاعتماد على الدعم الدولي لأصحاب المصلحة، من أجل تحقيق أهدافه في القضاء على المرض وطنيا وعالميا.

Translated from English version into Arabic by Lima SM and Dima Shs, through

TRANSLATORS
WITHOUT BORDERS

缅甸淋巴丝虫病分布及其系统消除行动进展（2000–2014）

Ni Ni Aye, Zaw Lin, Khin Nan Lon, Nay Yi Yi Linn, Thet Wai Nwe, Khin Mon Mon, Kapa Ramaiah, Hannah Betts, Louise A. Kelly-Hope

摘要

引言：在缅甸，淋巴丝虫病（LF）作为一种地方性流行疾病已被列入消除计划。为强调缅甸国家消除淋巴丝虫病项目（NPELF）进程，本文着重研究了缅甸 LF 的地理分布，大规模药物治疗（MDA）的影响和扩展，以及五个地区传播减弱的首要证据。

方法：根据 NPELF 收集的历史和基线流行数据，本研究绘制 LF 流行地图进一步确定 LF 分布情况。本研究还总结了 MDA 执行、报道覆盖率和哨点监测的数据；根据其现有流行数据制定统计模型，按测量年份预测乡镇级别流行率。此外，本研究使用传播评估调查（TAS）方法，测定儿童中抗原血症（Ag）的流行率，以确定目前 LF 流行率是否低于疾病不再复发的水平。

结果：经研究发现，中部谷区的基线 LF 的发病率最高。MDA 实施范围已扩大至 45 个地区，覆盖了大部分流行区，药物覆盖率达 60%–98.5%。但是在一些地区中，由于存在与药物供应和当地冲突有关的挑战，MDA 中断。总之，研究表明 LF 患病率明显降低，尤其是在相应模式支持下实施第 2 至 3 轮 MDA 之后，LF 患病率更为显著。 TAS 仅发现两名 Ag 阳性患儿，所以五个地区都通过了临界阈值。

结论：尽管面临诸多挑战，缅甸 NPELF 在消除 LF 中依然取得积极进展。但它需继续借助国际利益相关方的支持，保持良好进展态势，以实现本国和全球消除 LF 目标。

Translated from English version into Chinese by Qing-Yun Chen, edited by Jin Chen, through

CHINESE CENTER FOR DISEASE CONTROL AND PREVENTION
NATIONAL INSTITUTE OF PARASITIC DISEASES

Filariose lymphatique et progrès des activités d'élimination programmatique au Myanmar, 2000-2014

Ni Ni Aye, Lin Zaw, Khin Nan Lon, Nay Yi Yi Linn, Thet Wai Nwe, Khin Mon Mon, Kapa Ramaiah, Hannah Betts et Louise A. Kelly-Hope

RÉSUMÉ

Contexte : le Myanmar vise l'élimination de la filariose lymphatique, qui est endémique sur son territoire.Afin de mettre en lumière les progrès du Programme national pour l'élimination de la filariose lymphatique (NPELF) entre 2000 et 2014, le présent article décrit la répartition géographique de la filariose lymphatique, la montée en puissance et l'impact de la distribution de masse de médicaments (DMM), ainsi que les premiers signes d'un ralentissement de la transmission dans cinq districts.
Méthodes : la distribution de la filariose lymphatique a été déterminée en cartographiant les données de prévalence historiques et initiales recueillies dans le cadre du NPELF. Les données sur le déploiement des distributions de masse de médicaments, les taux de couverture rapportés et la surveillance des sites sentinelles sont rapportés. Un modèle statistique a été développé à partir des données de prévalence disponibles pour prédire la prévalence au niveau des villages selon l'année de la mesure. Les méthodes d'enquête d'évaluation de la transmission (TAS) par la mesure de la prévalence de l'antigénémie (Ag) chez les enfants ont été utilisées pour déterminer si la prévalence était inférieure à un seuil en dessous duquel une recrudescence était peu probable.
Résultats : la prévalence initiale la plus élevée de la filariose lymphatique a été relevée dans la région des vallées centrales. Les activités de distribution de masse de médicaments ont été étendues à 45 districts, soit la majorité de la population affectée par une filariose endémique, avec un taux de couverture médicamenteuse compris entre 60 % et 98,5 %. Des difficultés ont été rapportées en relation avec l'approvisionnement en médicaments et les conflits locaux, qui ont interrompu la DMM dans certains districts. Dans l'ensemble, on a constaté une baisse significative de la prévalence de la filariose lymphatique, notamment après les 2 ou 3 premières distributions, comme l'a confirmé le modèle correspondant. Les enquêtes d'évaluation de la transmission n'ont trouvé que deux enfants dont l'antigénémie était positive, ce qui signifie que les cinq districts sont passés en dessous du seuil critique.
Conclusion : dans l'ensemble, malgré plusieurs difficultés, le NEPLF du Myanmar a progressé vers l'élimination de la filariose lymphatique. Il faut à présent continuer sur cet élan, en profitant du soutien des décideurs internationaux, afin de progresser vers les objectifs nationaux et mondiaux d'élimination de la maladie.

Translated from English version into French by Susanne Assenat and Ode Laforge, through

TRANSLATORS
WITHOUT BORDERS

Лимфатический филяриоз и ход реализации программных мероприятий по его ликвидации в Мьянме в период с 2000 по 2014 гг.

Ни Ни Айе, Зау Лин, Кхин Нах Лон, Най Йи Йи Линн, Тхет Вай Нве, Кхин Мон Мон, Капа Рамайах, Ханна Беттс, Луиз А. Келли-Хоуп

РЕФЕРАТ

Справочная информация: Лимфатический филяриоз (ЛФ) в Мьянме носит эндемический характер и подлежит ликвидации. Чтобы обозначить прогресс Государственной программы по ликвидации лимфатического филяриоза (ГПЛЛФ) в период с 2000 по 2014 гг., в настоящей работе акцентируется внимание на географическом распределении указанного заболевания, на расширении масштабов, а также на последствиях внедрения массового применения лекарств (МПЛ), равно как и на первых свидетельствах сокращения передачи инфекции в пяти районах страны.

Методы: Определение распространения ЛФ было произведено с помощью отображения полученных в рамках ГПЛЛФ данных об историческом и базовом уровнях заболеваемости. Данные о внедрении МПЛ, о заявленном уровне охвата, а также о дозорном эпидемиологическом надзоре были представлены в краткой форме. На основе данных об уровне заболеваемости была разработана статистическая модель прогнозирования заболеваемости на поселковом уровне по году измерения. Для выявления, находился ли показатель заболеваемости ниже уровня, при котором обострение заболевания маловероятно, задействовалась методика обследования по оценке передачи (TAS), измеряющая преобладание антигенемии у детей.

Результаты: Наиболее высокий базовый уровень заболеваемости ЛФ был обнаружен в регионе Центральной долины. Масштаб мероприятий по внедрению МПЛ был расширен с целью охвата 45 районов, в которых проживает большинство эндемичного населения, а диапазон уровня охвата лекарственными препаратами составил от 60% до 98,5%. Сообщалось о проблемах, связанных как с поставками лекарственных препаратов, так и с местными конфликтами, что приводило к прерыванию МПЛ в некоторых районах. В целом, наблюдалось значительное снижение заболеваемости ЛФ, особенно после 2 или 3 циклов МПЛ, что подтверждалось соответствующей моделью. Применение методики TAS выявило всего два положительных случая антигенемии у детей, что позволило пересечь критический порог во всех пяти районах.

Заключение: В целом, несмотря на ряд проблем, Государственная программа по ликвидации лимфатического филяриоза в Мьянме продвинулась вперёд в устранении ЛФ, тем не менее, необходимо сохранять динамику, опираться на поддержку международных заинтересованных сторон, а также стремиться к достижению целей по ликвидации заболевания как на государственном, так и на международном уровнях.

Translated from English version into Russian by Liudmila Tomanek and Ekaterina Rugg, through

TRANSLATORS
WITHOUT BORDERS

La filariasis linfática y el progreso de las actividades de erradicación programada en Birmania del año 2000 al 2014

Ni Ni Aye Zaw Lin, Khin Nan Lon, Nay Yi Yi Linn, Thet Wai Nwe, Khin Mon Mon, Kapa Ramaiah, Hannah Betts, Louise A. Kelly-Hope

RESUMEN

Introducción: la filariasis linfática (FL) es endémica en Birmania y se pretende erradicar. Para resaltar el avance del programa nacional para erradicar la filariasis linfática (NPELF, por sus siglas en inglés) entre el año 2000 y 2014, este artículo destaca la distribución geográfica de la FL, la expansión y el impacto de la implementación de la administración masiva de medicamentos (AMM), y la primera evidencia de la disminución de la transmisión en cinco distritos.

Métodos: Se determinó la distribución de la FL cartografiando datos de prevalencia histórica y basal recabados por el NPELF. Se presentaron datos de la implementación de la AMM, los índices de cobertura notificados y la vigilancia del centro centinela. A partir de los datos de prevalencia disponibles se desarrolló un modelo estadístico para predecir la prevalencia a nivel municipal en cada año de medición. Se emplearon métodos de encuestas de evaluación de la transmisión (EET), con determinación de la prevalencia del antígeno (Ag) en sangre en niños, para determinar si la prevalencia era inferior al nivel con el que es improbable que haya un recrudecimiento.
Resultados: la mayor prevalencia basal de FL se observó en la región del Valle Central. La implementación de actividades de AMM se amplió hasta cubrir 45 distritos, que representan la mayor parte de la población endémica, con índices de cobertura farmacológica del 60 % al 98,5 %. Se comunicaron problemas relativos al suministro de fármacos y conflictos locales, y en algunos distritos se interrumpió la AMM. En general, se observaron reducciones considerables en la prevalencia de FL, especialmente después de las 2 o 3 primeras rondas de AMM, lo que respaldó el modelo correspondiente. Las actividades de EET solo detectaron dos niños con Ag positivos, por lo que los cinco distritos superaron el umbral crítico.
Conclusión: en general, a pesar de diversos problemas, el NPELF de Birmania ha dado pasos positivos para la erradicación de la FL. Sin embargo, es necesario mantener el impulso recurriendo al apoyo de colaboradores internacionales, para avanzar hacia los objetivos de erradicación nacional y mundial.

Translated from English version into Spanish by Sione Guitart and Astrid Walter Medina, through

TRANSLATORS
WITHOUT BORDERS
