# Supplementary material for: Mapping and modelling the impact of mass drug adminstration on filariasis prevalence in Myanmar
Source: Infect Dis Poverty. 2018 May 31;7:56. doi: 10.1186/s40249-018-0420-9 (PMC5984392; doi:10.1186/s40249-018-0420-9)
Supplement: Supplementary file 2 — Baseline Mf prevalence in district sentinel sites. (DOCX 35 kb) [file 40249_2018_420_MOESM2_ESM.docx]

**Additional file 1. Baseline Mf prevalence in district sentinel sites**

| **District#** | **State/Region** | **District** | **Sentinel site 1,2** | **Mf rate (%)** |
| --- | --- | --- | --- | --- |
| 1 | Magway | Magway 1 | Chauk/ward 1 | 7.1 / 1.1 |
|  | Magway | Magway 2 | Myo Thit/Myolulin | 1.18 |
| 2 | Magway | Thayet 1 | Thayet/Pyitawaye | 6.3 |
|  | Magway | Thayet 2 | Kan Ma/Pahto | 0.39 |
| 3 | Magway | Minbu 1 | Minbu/Kyauttan | 4.3 |
|  | Magway | Minbu 2 | Salin/Sinphyukyun | 3.03 |
| 4 | Magway | Pakokku 1 | Pakokku/Kanma | 0.19 |
|  | Magway | Pakokku 2 | Yaesakyo/Ward 8 | 9.1 |
| 5 | Sagaing | Sagaing 1 | Sagaing/Ayemyawaddy | 7.0 |
|  | Sagaing | Sagaing 2 | Sagaing/Nyaungpinwin | 3.0 |
| 6 | Sagaing | Monywa 1 | Monywa/Ayethayar | 5.5 |
|  | Sagaing | Monywa 2 | Ayartaw/Min village | 5.2 |
| 7 | Sagaing | Shwebo 1 | Taze/Pawu Bomya | 15.1 |
|  | Sagaing | Shwebo 2 | Depeyin/Saipyin | 11.5 |
| 8 | Sagaing | Katha 1 | Katha/Ward 1 | 0.53 |
|  | Sagaing | Katha 2 | Indaw/Naba villge | 2.6 |
| 9 | Sagaing | Kalay 1 | Kalay/Tar Han | 0 |
|  | Sagaing | Kalay 2 | Kalaywa |  |
| 10 | Sagaing | Tamu 1 | Tamu/Santagu | 0 |
|  | Sagaing | Tamu 2 | Tamu/Khan Pat | 1.0 |
| 11 | Mandalay | Mandalay 1 | Mandalay/Maharaungmyay | 0.7 |
|  | Mandalay | Mandalay 2 | Amarapura/Tharlayswa | 6.8 |
| 12 | Mandalay | Pyin OoLwin 1 | Pyin OoLwin/Ward 1 | 0.4 |
|  | Mandalay | Pyin OoLwin 2 | Mattaya/Kanphyu village | 0.2 |
| 13 | Mandalay | Kyauk Se 1 | Myitthar/Kume village | 5.47 |
|  | Mandalay | Kyauk Se 2 | Tatdar Oo/ward 1 | 14.7 |
| 14 | Mandalay | Ya methin 1 | Tat Kone/Sayarsan | 6 |
|  | Mandalay | Ya methin 2 | Le way/naung Bo village | 2.9 |
| 15 | Mandalay | Myin Gyan 1 | Kyautpadaung/Daung le village | 13.3 |
|  | Mandalay | Myin Gyan 2 | Nehtogyi/Tanzin village | 3.4 |
| 16 | Mandalay | Meikhtilar 1 | Won Twin/Ward 1 | 7.2 |
|  | Mandalay | Meikhtilar 2 | Tharsi/Nyaungyan village | 1.67 |
| 17 | Mandalay | Nyaung Oo 1 | Nyaung Oo/Taungpa village | 2.8 |
|  | Mandalay | Nyaung Oo 2 | Nyaung Oo/Tharyar Waddy | 7.4 |
| 18 | Rakhine | Sittwe 1 | Sittwe/Ka Thae | 12.6 |
|  | Rakhine | Sittwe 2 | Kyaut Taw/Shanywar | 1.4 |
| 19 | Rakhine | Maungdaw 1 | Maungdaw/Kyain Chaung | 0.16 |
|  | Rakhine | Maungdaw 2 | Buthetaung/Ward 1 | 1.7 |
| 20 | Rakhine | Kyauk Phyu 1 | Kyauk Phyu/ Pite Sait | 2.34 |
|  | Rakhine | Kyauk Phyu 2 | Kyauk Phyu/Theban Chaung |  |
| 21 | Rakhine | Thandwe 1 | Thanddwe/Kin Maw | 0 |
|  | Rakhine | Thandwe 2 | Thanddwe/Ward 1 |  |
| 22 | Chin | Paletwa 1 | Paletwa/Yeikkhar | 1.38 |
|  | Chin | Paletwa 2 | Paletwa/natmadar village | 1.59 |
| 23 | Mon | Mawlamyaing 1 | Mawlamyaing/Tharyaraye | 2.5 |
|  | Mon | Mawlamyaing 2 | Thanphyuzeyat/Aungchanthar | 0.83 |
| 24 | Mon | Thaton 1 | Thaton/Lateinn | 3.2 |
|  | Mon | Thaton 2 | Beelin/Kantharyar | 0.8 |
| 25 | Bago | Bago 1 | Bago/Shin Saw Pu | 0.4 / 0.0 |
|  | Bago | Bago 2 | Kyauttakhar/ Post Ward | 2.8 |
| 26 | Bago | Taung Ngu 1 | Taung Ngu/Ward 19 | 0.76 / 0.19 |
|  | Bago | Taung Ngu 2 | Pyhyuu/South Station | 1.18 |
| 27 | Bago | Thayawaddy 1 |  | 0 |
| 28 | Bago | Pyay 1 | Pyay/na Win | 0.59 / 0.39 |
|  | Bago | Pyay 2 | Pa Taung/ Won Lo | 0.58 |
| 29 | Ayeyarwaddy | Pathein 1 | Pathein/ Ward 6 | 2.4 / 0 |
|  | Ayeyarwaddy | Pathein 2 | Yekyi |  |
| 30 | Ayeyarwaddy | Henzada 1 | Zalon | 1.43 |
|  | Ayeyarwaddy | Henzada 2 | Myaung Aung | 0 |
| 31 | Ayeyarwaddy | Myaungmya 1 | Myaungmya/Ward | 0.59 |
|  | Ayeyarwaddy | Myaungmya 2 | Wakema |  |
| 32 | Ayeyarwaddy | Phyarpone 1 | Bokalay | 0 / 0 |
|  | Ayeyarwaddy | Phyarpone 2 | Daedaye | 0.8 |
| 33 | Ayeyarwaddy | Maubin 1 | Maubin | 0 / 0 |
|  | Ayeyarwaddy | Maubin 2 | Danubyu |  |
| 34 | Tanintharyi | Dawei 1 | Dawei/Kyatsarpin | 1.8 / 0.6 |
|  | Tanintharyi | Dawei 2 | Dawei/North kayatpyin | 0.59 |
| 35 | Tanintharyi | Myeik 1 | Myeik/Kan Gaung | 0.4 |
|  | Tanintharyi | Myeik 1 | Myeik/Myit Nge | 0 |
| 36 | Tanintharyi | Myeik 2 | Tanintharyi/Chaung Gyi | 0.2 |
|  | Tanintharyi | Kawthaung 1 | Kawthaung/Shwe Sin Yaw | 0.4 |
|  | Tanintharyi | Kawthaung 2 | Boat Pyin/ Aye Chan Thar | 0.2 |
| 37 | Kachin | Myitkyina 1 | Currently being conducted |  |
| 38 | Kachin | Bammaw 1 | Currently being conducted |  |
| 39 | Kayin | Hpaan 1 | Hpaan/Ward 5 | 0 |
|  | Kayin | Hpaan 2 | Hlaing bwe/ Ward D | 0 |
| 40 | Kayin | Kawkareik 1 | Kawkareik/Ward 7 | 0 |
|  | Kayin | Kawkareik 2 | Pyarinnseilgyi/Ward 3 | 0 |
| 41 | Kayin | Myawaddy 1 | Myawaddy/Ward 3 | 0.1 |
| 42 | Yangon | Yangon East 1 | Daw Pon/ Thinbawkyin | 0 |
|  | Yangon | Yangon East 2 | South Dagon/Ward 25 | 0 |
| 43 | Yangon | Yangon West 1 | Hlaing/Ward 16 | 0 |
|  | Yangon | Yangon West 2 | Alone/Sin Min | 0.1 |
| 44 | Yangon | Yangon North 1 | Hlaing Thar Yar/Ward 7 | 0 |
|  | Yangon | Yangon North 2 | Insein/Taungthukone | 0 |
| 45 | Yangon | Yangon South 1 | Dala/Bayinnaung | 0.4 |
|  | Yangon | Yangon South 2 | Tontae/Pyawbawle | 0.4 |

Note. Blank cells indicate where a sentinel site was identified but no survey conducted prior to MDA or are currently being conducted
