# Supplementary material for: Mapping and modelling the impact of mass drug adminstration on filariasis prevalence in Myanmar
Source: Infect Dis Poverty. 2018 May 31;7:56. doi: 10.1186/s40249-018-0420-9 (PMC5984392; doi:10.1186/s40249-018-0420-9)
Supplement: Supplementary file 3 — Matrix of MDA in endemic districts from 2001 to 2014. (DOCX 37 kb) [file 40249_2018_420_MOESM3_ESM.docx]

**Additional file 2. Matrix of MDA in endemic districts from 2001-2014**

| **Region/ State** | **No** | **District** | **2001** | **2002** | **2003** | **2004** | **2005** | **2006** | **2007** | **2008** | **2009** | **2010** | **2011** | **2012** | **2013** | **2014** | **Total**  **No.**  **MDAs** |
| --- | --- | --- | --- | --- | --- | --- | --- | --- | --- | --- | --- | --- | --- | --- | --- | --- | --- |
| Magway Region | 1 | Magway # | 1 | 1 | 1 | 1 | No  MDA | 1 | 1 | No  MDA | 1 | 1 | 1 | No  MDA | 1 | 1 | 11 |
|  | 2 | Thayet# | 1 | 1 | 1 | 1 |  | 1 | 1 |  | 1 | 1 | 1 |  | 1 | 1 | 11 |
|  | 3 | Minbu# | x | 1 | 1 | 1 |  | 1 | 1 |  | 1 | 1 | 1 |  | 1 | 1 | 10 |
|  | 4 | Pakokku# | × | 1 | 1 | 1 |  | 1 | 1 |  | 1 | 1 | 1 |  | 1 | 1 | 10 |
| Sagaing Region | 5 | Sagaing# | × | 1 | 1 | 1 |  | 1 | 1 |  | 1 | 1 | 1 |  | 1 | 1 | 10 |
|  | 6 | Monywa# | × | 1 | 1 | 1 |  | 1 | 1 |  | 1 | 1 | 1 |  | 1 | 1 | 10 |
|  | 7 | Shwebo# | × | 1 | 1 | 1 |  | 1 | 1 |  | 1 | 1 | 1 |  | 1 | 1 | 10 |
|  | 8 | Katha# | × | 1 | 1 | 1 |  | 1 | 1 |  |  |  |  |  |  |  | 5 |
|  | 9 | Kalay# | × | 1 | 1 | 1 |  | 1 | 1 |  |  |  |  |  |  |  | 5 |
|  | 10 | Tamu# | × | 1 | 1 | 1 |  | 1 | 1 |  |  |  |  |  |  |  | 5 |
| Mandalay Region | 11 | Mandalay | × | × | × | 1 |  | × | 1 |  | 1 | × | 1 |  | 1 | 1 | 6 |
|  | 12 | Pyin Oo Lwin | × | × | × | 1 |  | × | 1 |  | 1 | × | 1 |  | 1 | 1 | 6 |
|  | 13 | Kyauk Se | × | × | × | 1 |  | × | 1 |  | 1 | × | 1 |  | 1 | 1 | 6 |
|  | 14 | Ya Methin | × | × | × | 1 |  | × | 1 |  | 1 | × | 1 |  | 1 | 1 | 6 |
|  | 15 | Myin Gyan | × | × | × | 1 |  | × | 1 |  | 1 | × | 1 |  | 1 | 1 | 6 |
|  | 16 | Meikhtilar | × | × | × | 1 |  | × | 1 |  | 1 | × | 1 |  | 1 | 1 | 6 |
|  | 17 | Nyaung Oo | × | × | × | 1 |  | × | 1 |  | 1 | × | 1 |  | 1 | 1 | 6 |
|  |  | Nay Pyi Taw** | x | x | x | x |  | x | x |  | x | x | 1 |  | 1 | 1 | 3 |
| Rakhine State | 18 | Sittwe# | × | × | × | 1 |  | 1 | 1 |  | 1 | 1 | 1 |  | x | x | 6 |
|  | 19 | Maungdaw# | × | × | × | 1 |  | 1 | 1 |  | 1 | 1 | 1 |  | x | x | 6 |
|  | 20 | Kyauk Phyu# | × | × | × | 1 |  | 1 | 1 |  | 1 | 1 | 1 |  | x | x | 6 |
|  | 21 | Thandwe# | × | × | × | 1 |  | 1 | 1 |  | 1 | 1 | 1 |  | x | x | 6 |
| Chin State | 22 | Paletwa# | × | × | × | 1 |  | 1 | 1 |  | 1 | 1 | 1 |  | x | 1 | 7 |
| Mon State | 23 | Mawlamyaing | × | × | × | × |  | × | × |  | × | × | × |  | 1 | 1 | 2 |
|  | 24 | Thaton* | × | × | × | × |  | × | × |  | × | × | × |  | 1 | 1 | 2 |
| Bago Region | 25 | Bago* | × | × | × | × |  | × | × |  | × | × | × |  | 1 | 1 | 2 |
|  | 26 | Taung Ngu* | × | × | × | × |  | × | × |  | × | × | × |  | 1 | 1 | 2 |
|  | 27 | Thayawaddy* | × | × | × | × |  | × | × |  | × | × | × |  | 1 | 1 | 2 |
|  | 28 | Pyay* | × | × | × | × |  | × | × |  | × | × | × |  | 1 | 1 | 2 |
| Ayeyawaddy Region | 29 | Pathine* | × | × | × | × |  | × | × |  | × | × | × |  | 1 | 1 | 2 |
|  | 30 | Henzada* | × | × | × | × |  | × | × |  | × | × | × |  | 1 | 1 | 2 |
|  | 31 | Myaungmya* | × | × | × | × |  | × | × |  | × | × | × |  | 1 | 1 | 2 |
|  | 32 | Phyarpone* | × | × | × | × |  | × | × |  | × | × | × |  | 1 | 1 | 2 |
|  | 33 | Maubin* | × | × | × | × |  | × | × |  | × | × | × |  | 1 | 1 | 2 |
| Taninthayi Region | 34 | Dawei* | × | × | × | × |  | × | × |  | × | × | × |  | 1 | 1 | 2 |
|  | 35 | Myeik* | × | × | × | × |  | × | × |  | × | × | × |  | 1 | 1 | 2 |
|  | 36 | Kawthaung* | × | × | × | × |  | × | × |  | × | × | × |  | 1 | 1 | 2 |
| Kachin State | 37 | Myitkyina## | × | × | × | × |  | × | × |  | × | × | × |  | x | x | 0 |
|  | 38 | Bammaw## | × | × | × | × |  | × | × |  | × | × | × |  | x | x | 0 |
| Kayin State | 39 | Hpaan* | × | × | × | × |  | × | × |  | × | × | × |  | 1 | 1 | 2 |
|  | 40 | Kawkareik* | × | × | × | × |  | × | × |  | × | × | × |  | 1 | 1 | 2 |
|  | 41 | Myawaddy | × | × | × | × |  | × | × |  | × | × | × |  | 1 | 1 | 2 |
| Yangon Region | 42 | Yangon East* | × | × | × | × |  | × | × |  | × | × | × |  | 1 | 1 | 2 |
|  | 43 | Yangon West* | × | × | × | × |  | × | × |  | × | × | × |  | 1 | 1 | 2 |
|  | 44 | Yangon North* | × | × | × | × |  | × | × |  | × | × | × |  | 1 | 1 | 2 |
|  | 45 | Yangon South* | × | × | × | × |  | × | × |  | × | × | × |  | 1 | 1 | 2 |
|  |  |  | 2 | 10 | 10 | 22 |  | 15 | 22 |  | 19 | 12 | 1  9 |  | 36 | 37 |  |

Notes for table.

* Significant up-scaling of programme and first round of MDA in 2013

** Nay Pyi Taw a newly formed territory in 2005, part of Mandalay Region and in 2013 started to be administered separately

# Down-scaling of the programme possible during next 1-2 years

## MDA has not been scaled up or has been stopped due to security concerns

Green – stopped due to low Mf and conducted cluster survey equivalent to TAS 1 using WHO guidelines
