# Supplementary material for: Mapping and modelling the impact of mass drug adminstration on filariasis prevalence in Myanmar
Source: Infect Dis Poverty. 2018 May 31;7:56. doi: 10.1186/s40249-018-0420-9 (PMC5984392; doi:10.1186/s40249-018-0420-9)
Supplement: Supplementary file 4 — ICT Survey in school children in Tamu Township District in 2008. (DOCX 23 kb) [file 40249_2018_420_MOESM4_ESM.docx]

**Additional file 3. ICT Survey in school children in Tamu Township District in 2008**

| **S/N** | **Name of School** | **No. of school children tested with ICT** | **No. Positive** |
| --- | --- | --- | --- |
| 1 | Boke Kan (Primary School) | 73 | 0 |
| 2 | Khan Pat Primary School | 183 | 0 |
| 3 | Kanmagyi (Post primary school) | 60 | 0 |
| 4 | TinZin Primary School | 15 | 0 |
| 5 | Khanpat (East) Primary School | 57 | 0 |
| 6 | Pantha Primary School | 85 | 0 |
| 7 | Htantapin Primary School | 58 | 0 |
| 8 | Indine Primary School | 33 | 0 |
| 9 | Khameik Primary School | 43 | 0 |
| 10 | Nanmonta Primary School | 21 | 0 |
| 11 | Yanlinpine Primary School | 29 | 0 |
| 12 | Witoke Primary School | 105 | 0 |
| 13 | Minthamee Primary School | 68 | 0 |
| 14 | Kanan Primary School | 199 | 0 |
| 15 | Khanpat State High school No.(1) | 238 | 0 |
| 16 | Khanpat State High school No.(2) | 207 | 0 |
| 17 | Nankataik Primary School | 40 | 0 |
| 18 | No.4 primary school, Tamu | 122 | 0 |
| 19 | State iddle School, Tamu | 331 | 0 |
| 20 | No.1 primary school, Tamu | 396 | 0 |
| 21 | No.5 primary school, Tamu | 238 | 0 |
| 22 | No.2 primary school, Tamu | 113 | 0 |
| 23 | No.9 primary school, Tamu | 119 | 0 |
| 24 | Nanphalon Primary school | 200 | 0 |
| 25 | He Zin primary school | 52 | 0 |
|  |  | **3085** |  |

Note: Another ICT survey in 2008 using a cluster sampling strategy to test 300 children age 2 to 4 years of age

also found all children negative.
