# Supplementary material for: Mapping and modelling the impact of mass drug adminstration on filariasis prevalence in Myanmar
Source: Infect Dis Poverty. 2018 May 31;7:56. doi: 10.1186/s40249-018-0420-9 (PMC5984392; doi:10.1186/s40249-018-0420-9)
Supplement: Supplementary file 5 — ICT Survey in school children in Katha District in 2008. (DOCX 25 kb) [file 40249_2018_420_MOESM5_ESM.docx]

| **Additional file 4. ICT Survey in school children in Katha District in 2008** | | | | | | | | | | |  |  |
| --- | --- | --- | --- | --- | --- | --- | --- | --- | --- | --- | --- | --- |
|  |  |  | |  |  |  |  |  |  |  |  |  |
| **Township** | **S/N** | **Name of School** | | **No. of School Children ICT tested** | **No. Positive** | | | |  |  |  |  |
| Katha | 1 | Katha SPS | | 141 | 0 | | | |  |  |  |  |
|  | 2 | Aug Waegyi SPS | | 84 | 0 | | | |  |  |  |  |
|  | 3 | Chaung Wa SPS | | 143 | 0 | | | |  |  |  |  |
|  | 4 | Pyi Htaung Lay SPS | | 187 | 0 | | | |  |  |  |  |
|  | 5 | Ah Lae Kyun SPS | | 97 | 0 | | | |  |  |  |  |
|  | 6 | Set SokeSPS | | 102 | 0 | | | |  |  |  |  |
| Indaw | 7 | Mae Zar SHS(B) | | 120 | 0 | | | |  |  |  |  |
|  | 8 | Myay Pa Lin SPS | | 49 | 0 | | | |  |  |  |  |
|  | 9 | Naung Moe SPS | | 17 | 0 | | | |  |  |  |  |
|  | 10 | Nga Pyay Inn SPS | | 34 | 0 | | | |  |  |  |  |
| Wuntho | 11 | Swbwagyi SPS | | 66 | 0 | | | |  |  |  |  |
|  | 12 | Pan Teingone SPS | | 35 | 0 | | | |  |  |  |  |
| Kawlin | 13 | Kawlin SMS | | 281 | 0 | | | |  |  |  |  |
|  | 14 | Innma SPS | | 48 | 0 | | | |  |  |  |  |
|  | 15 | Khamauk Taung SMS | | 140 | 0 | | | |  |  |  |  |
|  | 16 | Singone | | 100 | 0 | | | |  |  |  |  |
|  | 17 | Intapin | | 131 | 0 | | | |  |  |  |  |
|  | 18 | Chaung Na | | 128 | 0 | | | |  |  |  |  |
|  | 19 | Shan Kalone | | 69 | 0 | | | |  |  |  |  |
| Pinlebu | 20 | Michaung Inn | | 152 | 0 | | | |  |  |  |  |
|  | 21 | Naung Yin | | 68 | 0 | | | |  |  |  |  |
|  | 22 | Sin Laung | | 64 | 0 | | | |  |  |  |  |
|  | 23 | Tamaw thar | | 40 | 0 | | | |  |  |  |  |
| Ban Mauk | 24 | Myoma (2) | | 52 | 0 | | | |  |  |  |  |
|  | 25 | Aung Thagone | | 68 | 0 | | | |  |  |  |  |
|  | 26 | Kyun Taw | | 130 | 0 | | | |  |  |  |  |
|  | 27 | Nantpon | | 42 | 0 | | | |  |  |  |  |
| Htee Chaing | 28 | Nyaungpintha | | 201 | 0 | | | |  |  |  |  |
|  | 29 | Sin Kyun | | 83 | 0 | | | |  |  |  |  |
|  | 30 | Sittan | | 85 | 0 | | | |  |  |  |  |
|  | 31 | Wunlokone | | 46 | 0 | | | |  |  |  |  |
| **Total no. of primary school children** | | | | **3003** |  | | | |  |  |  |  |
|  |  | |  |  |  |  | | | |  |  |  |
| Note: SPS - State Primary School; SMS - State Middle School; SHS - State High School | | | | | | | | | | | |  |
| Although 30 Cluster schools were chosen, according to the No. of students attending at survey period, the last one had to be included in the survey to fulfil 3000 No. of children. | | | | | | | | | | | |  |
