# Supplementary material for: Mapping and modelling the impact of mass drug adminstration on filariasis prevalence in Myanmar
Source: Infect Dis Poverty. 2018 May 31;7:56. doi: 10.1186/s40249-018-0420-9 (PMC5984392; doi:10.1186/s40249-018-0420-9)
Supplement: Supplementary file 6 — ICT Survey in infant and school children from Kalay District in 2008. (DOCX 22 kb) [file 40249_2018_420_MOESM6_ESM.docx]

**Additional file 5. ICT Survey in infant and school children from Kalay District in 2008**

|  |  |  |  |  |
| --- | --- | --- | --- | --- |
| **Sr.No** | **Name of Cluster** | **2-4 Yr Children  ICT Test** | **5-6 Yr School  ICT Test** | **No. Positive** |
| 1 | Thanpho | 10 | 103 | 0 |
| 2 | Tinthar | 10 | 132 | 0 |
| 3 | Natmyaung | 10 | 91 | 0 |
| 4 | NawNyaungbin | 10 | 121 | 0 |
| 5 | Zinglin | 10 | 62 | 0 |
| 6 | Lechar | 10 | 72 | 0 |
| 7 | KyaungTike | 10 | 151 | 0 |
| 8 | Kyatphanet | 10 | 113 | 0 |
| 9 | Nansaungpu | 10 | 100 | 0 |
| 10 | Tahan Ward (No.5) | 10 | 0 | 0 |
| 11 | Chinsu | 10 | 45 | 0 |
| 12 | Letpanchaung | 10 | 196 | 0 |
| 13 | Aungthitsa Ward | 10 | 247 | 0 |
| 14 | Pinlon Ward | 10 | 570 | 0 |
| 15 | Kyoethonebin | 10 | 106 | 0 |
| 16 | Tahan Ward(No.7) | 10 | 0 | 0 |
| **Total** | | **160** | **2109** |  |
